# Assessment of asthma severity in adults with ever asthma: A continuous score

**DOI:** 10.1371/journal.pone.0177538

**Published:** 2017-05-18

**Authors:** Lucia Calciano, Angelo Guido Corsico, Pietro Pirina, Giulia Trucco, Deborah Jarvis, Christer Janson, Simone Accordini

**Affiliations:** 1 Unit of Epidemiology and Medical Statistics, Department of Diagnostics and Public Health, University of Verona, Verona, Italy; 2 Division of Respiratory Diseases, IRCCS “San Matteo” Hospital Foundation, University of Pavia, Pavia, Italy; 3 Institute of Respiratory Diseases, University of Sassari, Sassari, Italy; 4 Department of Public Health and Pediatrics, University of Turin, Turin, Italy; 5 National Heart & Lung Institute, Imperial College, London, United Kingdom; 6 Department of Medical Sciences, Uppsala University, Uppsala, Sweden; Forschungszentrum Borstel Leibniz-Zentrum fur Medizin und Biowissenschaften, GERMANY

## Abstract

**Background:**

In epidemiological studies, continuous measures of asthma severity should be used to catch the heterogeneity of phenotypes. This study aimed at developing and validating continuous measures of asthma severity in adult patients with ever asthma from the general population, to be used in epidemiological studies.

**Methods:**

Respiratory symptoms, anti-asthmatic treatment and lung function were measured on 520 patients with ever asthma aged 20–64 years from the general Italian population (GEIRD study; 2007/2010). The variables that represent the same dimension of asthma severity were identified through an exploratory factor analysis and were summarized through a multiple factor analysis.

**Results:**

Only respiratory symptoms and anti-asthmatic treatment were summarized in a continuous score (STS). STS ranges from 0 (no symptoms/treatment) to 10 (maximum symptom frequency and treatment intensity). STS was positively correlated with the Global Initiative for Asthma classification of asthma severity computed on the 137 cases with a doctor's diagnosis (Spearman’s coefficient = 0.61, p-value<0.0001) (concurrent validity). Furthermore, using a cohort of 1,097 European asthmatics (ECRHS II study; 1999/2002), increasing STS levels at baseline (1991/1993) were positively associated with long-term outcomes (hospitalization and lost workdays for breathing problems, asthma attack frequency and use of asthma controllers) (predictive validity). Finally, the STS scores computed from the GEIRD and ECRHS II data were comparable (Lin’s coefficient = 0.95, p-value<0.0001) (replication analysis).

**Conclusions:**

STS is a valid and replicable measure of asthma severity in adults, which could be used in association studies.

## Introduction

Asthma represents a global health problem because of its high morbidity [1] and the heavy socio-economic burden [2–5].

The identification of the level of asthma severity is crucial for treatment decisions in clinical practice and for patients’ characterization in epidemiological studies. In fact, asthma is not a single disease and its severity is characterized by different phenotypes that may result from different risk factors [6–8]. However, defining 'asthma severity' is not an easy task, mainly because of its heterogeneity and the lack of a worldwide consensus on its definition [9]. According to the 2002 and 2004 Global Initiative for Asthma (GINA) guidelines [10], a categorical classification of asthma severity, which is based on symptom frequency, lung function and treatment intensity has been used in several epidemiological studies [11–16]. The choice of this composite measure is justified by the fact that no single measures could accurately reflect heterogeneous phenotypes of asthma severity. However, continuous outcomes should be used for epidemiological purposes because any categorical classification of disease severity is biologically unsatisfactory for the majority of chronic diseases [17]. Moreover, individual pathophysiological characteristics of asthma severity are mainly measured on continuous or ordinal scales [9]. In addition, recognizing the nature of asthma as a continuum increases the power of a statistical analysis [18] and has the potential to reduce bias in the evaluation of risk factors for asthma [19].

The present study is aimed at developing and validating continuous measures of asthma severity that summarize the individual information on lung function, symptom frequency and treatment intensity in adult patients with ever asthma from the general population, to be used in epidemiological studies. To fulfill this purpose, the data from the Gene Environment Interactions in Respiratory Diseases (GEIRD) study were used.

## Methods

### Design of the GEIRD study

GEIRD (www.geird.org) is an ongoing multicentre, (multi)case-control study on respiratory health [20], which includes more than 20,000 subjects who were randomly selected from the general population in seven Italian centres (Ancona, Pavia, Salerno, Sassari, Terni, Turin and Verona). The cases of asthma, chronic obstructive pulmonary disease (COPD), chronic bronchitis and rhinitis, and the subjects without respiratory symptoms (controls) were identified through a two-stage screening process. In the first stage (2007–2010), the participants were administered a screening questionnaire on respiratory health. In the second stage (2008-ongoing), all the responders to the screening questionnaire with symptoms suggestive of asthma, COPD or chronic bronchitis, and a random sample of those with symptoms suggestive of rhinitis or without respiratory symptoms, were invited to undergo a detailed clinical interview, lung function and laboratory tests for accurate phenotyping. Ethics approval was obtained from the appropriate ethics committee (“Comitato Etico per la Sperimentazione dell'Azienda Ospedaliera Istituti Ospitalieri di Verona”). All participants were fully informed about all the aspects of the research project and they gave written informed consent.

### Cases of asthma

The subjects were defined as having asthma if they had reported at least one of the two following conditions at the clinical examination:

1. ever asthma;
2. asthma-like symptoms [wheezing, nocturnal tightness in the chest, shortness of breath (SoB) following strenuous activity, SoB at rest, SoB at night time] or anti-asthmatic treatment in the past 12 months at the clinical interview, and if they had fulfilled at least one of the following spirometric criteria:
  - being positive to the methacholine challenge test with a <1 mg dose producing a 20% fall in FEV_1_;
  - having a pre-bronchodilator airflow obstruction {FEV_1_/FVC <70% or <Lower Limit of Normal for FEV_1_/FVC according to Quanjer [21]} and a positive reversibility test (increase in FEV_1_ >12% and >200 ml with respect to pre-bronchodilator FEV_1_ after 400 mcg of salbutamol);
  - having pre- but not post-bronchodilator airflow obstruction, and a post-bronchodilator FEV_1_ ≥80% predicted [21].

**Fig 1:**
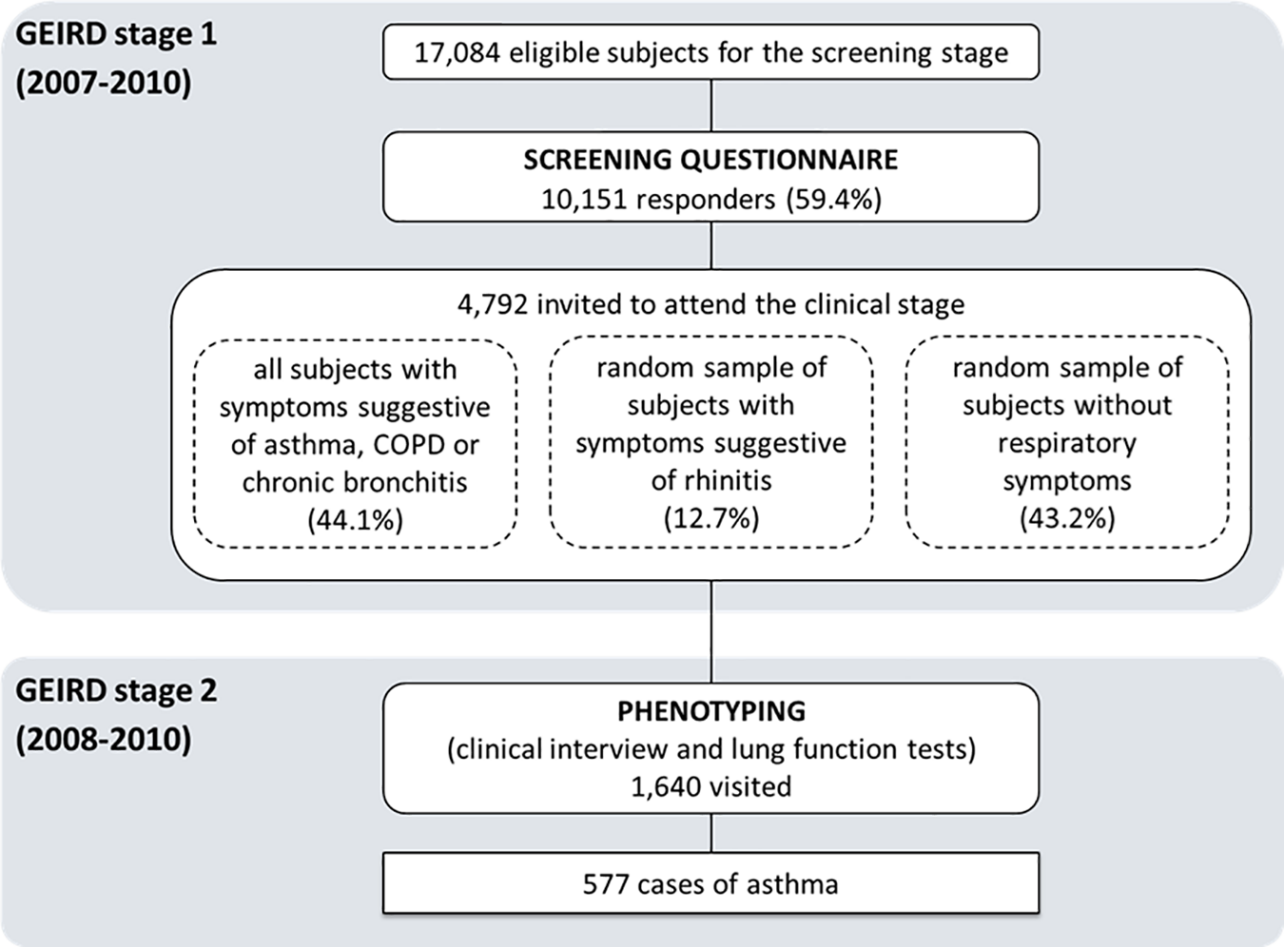
Flow chart of the GEIRD study in the Pavia, Sassari, Turin and Verona centres, and selection of the cases of asthma.

At the time of the present analysis, only the data on the patients recruited in the Pavia, Sassari, Turin and Verona centres were available. The screening questionnaire was mailed to 17,084 eligible subjects (GEIRD-stage 1; 2007–2010) in these centres. Among the responders (response rate: 59.4%), 4,792 subjects were invited for further clinical investigations (GEIRD-stage 2; 2008–2010). Among the 1,640 subjects who were phenotyped, 577 subjects were classified as cases of asthma (Fig 1).

### Computation of asthma severity scores

Asthma severity scores were devised through a two-step procedure, which was carried out on the 520 cases of asthma (out of the 577 cases identified in the four centres) with complete information on disease severity (respiratory symptoms, lung function and anti-asthmatic treatment).

#### Step 1

An exploratory factor analysis (EFA) [22] was performed on 11 variables (listed in Table 1) in order to identify the subset of variables representing the same dimension (factor) of disease severity. This model is based on the assumption that the symptom, treatment and lung function variables are correlated through some unobservable factors. The values of each variable were ordered coherently with an increasing level of disease severity. EFA was based on the mixed correlation matrix between each pair of variables [23], i.e. the polychoric correlation between two ordinal variables, the tetrachoric correlation between two dichotomous variables, the Pearson moment correlation between two continuous variables, and the polyserial correlation if one variable is categorical and the other one is continuous.

**Table 1:** Coding and definition of the candidate variables.

Name	Definition	Coding^†^
**Wheezing**	“How many times have you had wheezing or whistling in the last 12 months?”	0 (ever)
		1 (sometimes)
		2 (at least once a week)
**Asthma attacks**	“How many attacks of asthma have you had in the last 12 months?”	0 (none)
		1 (1–11 attacks)
		2 (≥12 attacks)
**Tightness in chest**	“Have you woken up with a feeling of tightness in your chest at any time in the last 12 months?”	0 (no)
		1 (yes)
**SOB at rest**	“Have you had an attack of shortness of breath that came on during the day when you were at rest at any time in the last 12 months?”	0 (no)
		1 (yes)
**SOB after strenuous activity**	“Have you had an attack of shortness of breath that came on following strenuous activity at any time in the last 12 months?”	0 (no)
		1 (yes)
**SOB at night time**	“Have you been woken by an attack of shortness of breath at any time in the last 12 months?”	0 (no)
		1 (yes)
**Chronic bronchitis**	Cough or phlegm on most days for a minimum of three months a year and for at least two successive years.	0 (no)
		1 (yes)
**Worsening of respiratory symptoms**	“In the last 12 months, have you had any episodes/times when your symptoms (cough, phlegm, shortness of breath) were a lot worse than usual?” or “In the last 12 months have you visited a hospital casualty department or emergency room (for breathing problems)?” or “In the last 12 months, have you spent a night in hospital (for breathing problems)?”	0 (no)
		1 (yes to at least one of the three questions)
**Treatment**	Intensity of anti-asthmatic treatment in the past 12 months.	0 (no treatment)
		1 (GINA step 1- only relievers)*
		2 (GINA step 1—controllers)**
		3 (GINA steps ≥2)***
**FEV**_**1**_**% predicted**	Pre-bronchodilator FEV_1_% predicted	(continuous)
**FEV**_**1**_**/FVC**	Pre-bronchodilator FEV_1_/FVC	(continuous)

^†^ all variables were ordered coherently with an increasing level of asthma severity, i.e. the higher the level of the observed variables, the higher the value of asthma severity.

* GINA step 1—only relievers: treatment with short-acting β2-agonists and/or anticholinergic and/or ketotifen without controllers in the past 12 months.

** GINA step 1—controllers with/without taking relievers: discontinuous treatment with Inhaled glucocorticosteroids (ICS) or Cromones or Oral Methylxanthines or Leukotriene modifiers or ICS & Long-acting β2-agonists or ICS & Methylxanthines or ICS & Leukotriene modifiers with/without taking relievers in the past 12 months.

*** GINA steps ≥2: treatment with ICS (daily) or Cromones (daily) or Oral Methylxanthines (daily) or Leukotriene modifiers (daily) or ICS & Long-acting β2-agonists (daily) or ICS & Methylxanthines (daily) or ICS & Leukotriene modifiers (daily) or Oral glucocorticosteroids or anti-IgE or Injective Corticosteroids with/without taking relievers in the past 12 months.

Factors were retained at EFA if the eigenvalue was greater than the mean of eigenvalues and by means of the break in the scree plot (eigenvalues vs. factors). The uniqueness (i.e. proportion of variance in a given variable that is not due to the extracted common factor) was used to identify the variables that were poorly correlated with the extracted factors. The Kaiser-Meyer-Olkin (KMO) measure was used for assessing the adequacy of the fitted model. The variables with a KMO values <0.7 were excluded from the later dimensionality reduction procedure (MFA).

#### Step 2

A multiple factor analysis (MFA) [24] was performed on the subset of variables representing each dimension of asthma severity (identified at EFA) in order to estimate the weights to be used for computing the corresponding individual score. In MFA, the symptom, treatment and lung function variables were grouped in three different sets. For each dimension of asthma severity, the individual scores were obtained as the weighted linear combination of the variables included in MFA (Methods section in S1 File). All variables were analyzed on the quantitative scale. Any component that had the eigenvalue greater than the mean of the eigenvalues was retained. Moreover, the break in the scree plot was used to retain components. A bootstrap procedure with 50,000 replications was used to obtain a stable solution for eigenvalues, weights and scores.

### Concurrent validity

Spearman's coefficients were computed to evaluate the concordance between the identified scores and a categorical classification of asthma severity, which was defined according to the GINA guidelines [25] on the 137 cases who had reported a doctor's diagnosis of asthma and complete information on the GINA classification of asthma severity.

### Predictive validity

The data from the European Community Respiratory Health Survey (ECRHS), which is an international, population based cohort study on respiratory health in subjects aged 20–44 years at the time of recruitment (ECRHS I; 1991–1993) [26], were used to evaluate the ability of the identified scores to predict future events. The predictive validity was verified on the subjects with ever asthma from 26 centres, who were identified at the ECRHS I and had participated in the ECRHS II (1999–2002), by evaluating the association between the scores computed at the ECRHS I (using the weights estimated from the GEIRD data) and the following long-term outcomes measured at the ECRHS II: (i) having at least one emergency department visit and/or hospital admission for breathing problems during the follow-up, (ii) number of asthma attacks in the past 12 months, (iii) use of asthma controllers in the past 12 months, and (iv) having lost at least one working day due to breathing problems in the past 12 months. The association between the ECRHS-scores and each outcome was evaluated by using two-level regression models, with subjects (1st level units) nested into centres (2nd level units). The hospitalization Rate Ratios (RR) were computed by using two-level Poisson regression models by setting the person-years equal to half the length of the follow-up in the case of at least one hospitalization. The adjusted Ratios of Expected number of asthma attacks (RE) were computed by using a two-level negative binomial regression model. We chose a negative binomial model because of the over-dispersed distribution of the “number of asthma attacks” variable (Pearson's moment coefficient of skewness = 8.7) and its better fit as compared to the Poisson model (likelihood ratio test, p-value<0.0001). The association of the ECRHS-scores with the remaining outcomes was measured through the adjusted Ratio of Expected values (RE) obtained by means of a two-level Poisson regression model. All the regression models had a random intercept term at the 2nd level, and the ECRHS-score and potential confounders (gender, age, BMI and smoking habits at baseline) as fixed effects. The 95% confidence intervals (CIs) were obtained by using a robust variance estimator (Huber-White sandwich estimator).

### Replicability

The robustness of the identified scores was verified by testing whether the MFA weights change when estimated from another, similar population [27]. The data from the subjects with ever asthma, who were identified at the ECRHS I and had participated in the ECRHS II, were used. The scores obtained by using the weights from the GEIRD data were compared to the scores of the same dimension, which were computed by using the weights from a new MFA on the ECRHS II data. The Lin’s concordance correlation coefficient was used (a value ≥0.80 indicates a good replicability).

The statistical analyses were carried out using STATA, version 13.0, and R version 3.1.0.

## Results

### Main characteristics of patients

In our sample, the percentage of females was 51.9%, the mean age was 43.0 years (SD, standard deviation; SD = 9.4) and the median BMI was 24.4 (IQR, interquartile range; IQR = 21.7–27.0) (Table 2). Ever smokers were predominant (51.4%). About two out of three patients reported at least one attack of asthma, the presence of at least one asthma-like symptom, the use of anti-asthmatic treatment, the worsening of symptoms or the use of hospital services in the past 12 months. In addition, 32.5% of these subjects had used anti-asthmatic treatment in the past 12 months, and 15.0% of the 314 asthmatics with available information had reported a not well controlled disease {i.e. 5-item Asthma Control Questionnaire (ACQ) score [28] less than 20}. Finally, pre-bronchodilator FEV_1_% predicted and FEV_1_/FVC were 102.2 (SD = 14.3) and 78.6 (SD = 7.8) on average, respectively.

**Table 2:** Main characteristics of the 520 cases of asthma identified in the GEIRD study.

Main characteristic	Sub-characteristic	Summary statistics	Numerical value
Females		%	51.9
Age (years)		mean ± sd	43.0 ± 9.4
Smoking habits	Never smoker	%	48.6
	Past smoker		26.0
	Current smoker		25.4
BMI		median (interquartile range)	24.4 (21.7–27.0)
Wheezing*	Never	%	60.0
	Sometimes		32.7
	At least once a week		7.3
Asthma attacks*	None	%	80.0
	1–11 attacks		15.6
	≥ 12 attacks		4.4
Tightness in chest*		%	21.0
SOB at rest*		%	12.1
SOB after strenuous activity*		%	23.3
SOB at night time*		%	16.0
Chronic bronchitis		%	17.7
Worsening of respiratory symptoms*		%	15.8
Treatment*	None	%	64.4
	GINA step 1—only relievers		14.0
	GINA step 1 –controllers		12.7
	GINA steps ≥ 2		8.9
Pre-bronchodilator FEV_1_% predicted		mean ± sd	102.2 ± 14.3
Pre-bronchodilator FEV_1_/FVC		mean ± sd	78.6 ± 7.8
ACOS**		%	3.1
ACQ	5–19	%	15.0
	20–24		38.2
	25		46.8

SOB, shortness of breath; ACOS, Asthma-COPD Overlap Syndrome; ACQ, Asthma Control Questionnaire.

* in the past 12 months.

** post-bronchodilator FEV_1_/FVC <Lower Limit of Normal according to Quanjer (21).

### Dimensionality reduction procedure

The mixed correlation matrix suggested that there was redundant information in the data and that the two lung function variables were weakly correlated with the variables regarding respiratory symptoms and anti-asthmatic treatment (Table A in S1 File). Moreover, the EFA identified one factor that explained 84% of the total variance, whereas this factor explained 5% and 8% of the variance of pre-bronchodilator FEV_1_% predicted and FEV_1_/FVC, respectively. In addition, the model adequacy checking indicates that the lung function variables may not belong to the extracted factor (Table B and Figure A in S1 File). Therefore, only the 9 variables regarding symptom frequency and anti-asthmatic treatment intensity represent the same dimension of asthma severity and were considered in the dimensionality reduction procedure.

The MFA extracted two components that accounted for 41% and 19% of the total variance, respectively. Only the first MFA component was considered as a score of asthma severity because all the weights had the positive sign, as expected for a measure of disease severity (Table C and Figure A in S1 File).

### Symptom frequency and anti-asthmatic Treatment intensity Score (STS)

According to the coding of the variables as reported in Table 1, the equation used to compute individual STS is the following:

STS=1.03(Wheezing)+0.85(Asthmaattacks)+0.48(Tightnessinchest)+0.32(SOBatrest)+0.46(SOBafterstrenuousactivities)+0.38(SOBatnighttime)+0.27(Chronicbronchitis)+0.34(Worseningofrespiratorysymptoms)+1.33(Treatment).

**Fig 2:**
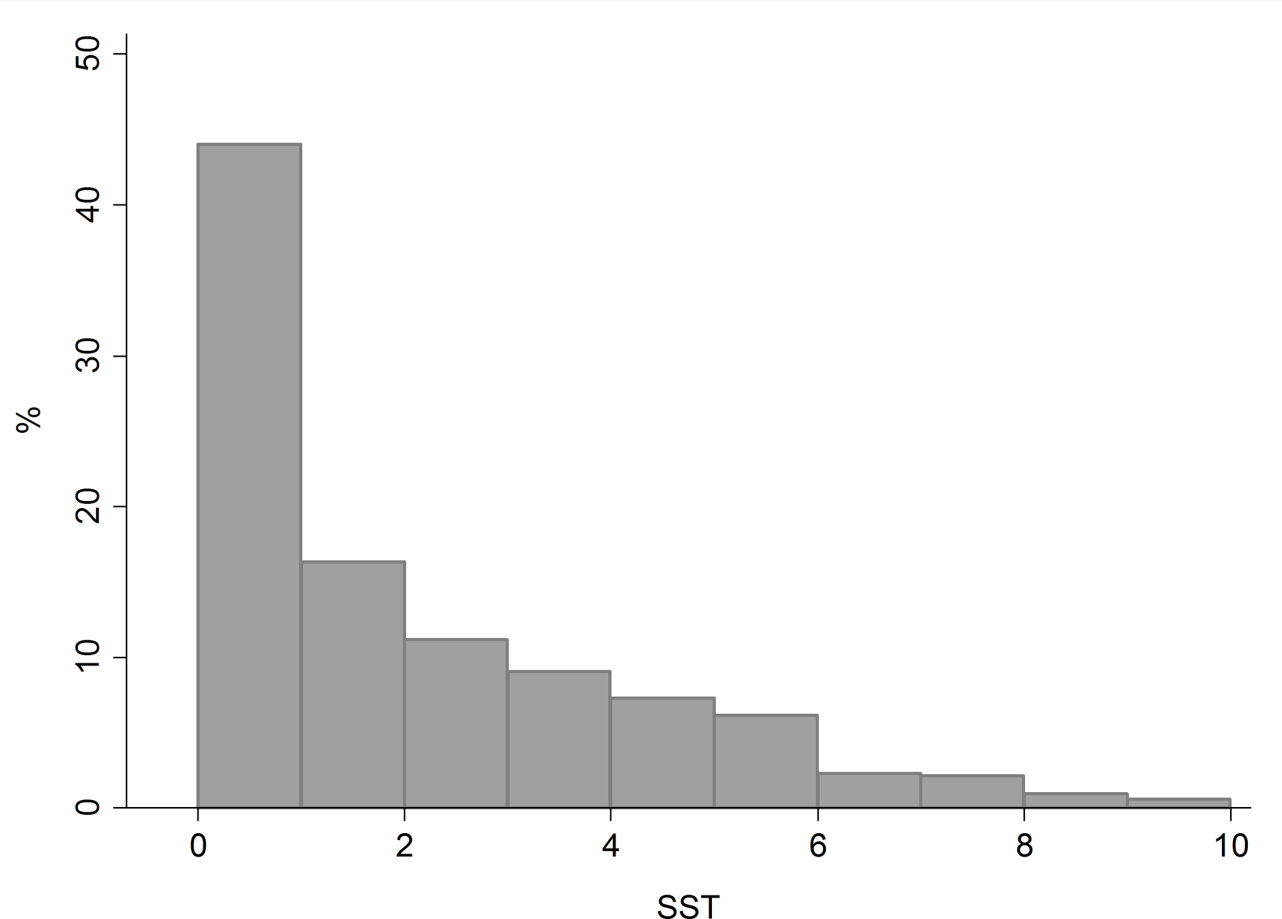
Distribution of the Symptom frequency and anti-asthmatic Treatment intensity Score (STS). Data from the GEIRD study.

STS ranges from 0 (no respiratory symptoms and no anti-asthmatic treatment) to 10 (maximum level of symptom frequency and treatment intensity) on a continuous scale, and it has a skewed distribution towards low values (Fig 2), which can be approximated to some common parametric distribution, such as a Zero-inflated Gamma. In our sample, STS ranged from 0 to 9.35 (median = 1.20, IQR = 0–3.53) and 33.5% of the 520 patients had a score equal to 0, because they had reported ever asthma without the presence of respiratory symptoms and without the use of asthma medications in the past 12 months.

### Concurrent validity of STS

Among the 137 GEIRD cases with an asthma diagnosis, the individuals who had received daily controller medications had a significantly lower STS (median = 0.46, IQR = 0–1.86) as compared to both the subjects treated with non-daily controller medications (median = 4.47, IQR = 3.48–5.47) and the subjects not treated with controller medications (median = 5.87, IQR = 4.45–7.47) in the past 12 months. Moreover, STS was positively correlated with the GINA classification of asthma severity (Spearman’s coefficient = 0.61, p-value<0.0001) (Table 3).

**Table 3:** Median of the Symptom frequency and anti-asthmatic Treatment intensity Score (STS) according to the GINA classification of asthma severity. Data from the GEIRD study.

GINA classification*	N	Median (IQR)	p-value
Intermittent	72	3.61 (2.58–4.48)	<0.00001^†^
mild persistent	17	5.17 (4.07–6.59)	
moderate persistent	17	5.66 (3.99–6.13)	
severe persistent	31	5.99 (5.02–7.70)	

IQR, interquartile range.

^*^ GINA classification was calculated on patients with a doctor’s diagnosis of asthma.

^†^ Spearman's correlation coefficient, which was computed to evaluate the concordance between STS and the GINA classification of asthma severity.

### Predictive validity of STS

In the ECRHS cohort, 1,097 subjects with ever asthma had provided complete information on STS at the ECRHS I and on at least one long-term outcome (Table D in S1 File). We found that the long-term outcomes were predicted by increased values of STS (Table 4). In particular, for one-unit increase in STS, the risk of hospitalization for breathing problems during the 9-year follow-up (between the ECRHS I and II) raised by 31% and the expected number of asthma attacks in the past 12 months at ECRHS II raised by 46%. Furthermore, at ECRHS II, the expected number of subjects with at least one working day lost due to breathing problems in the past 12 months increased by 25%; the same figure was observed for the use of controller medications.

**Table 4:** Association between the Symptom frequency and anti-asthmatic Treatment intensity Score (STS) at baseline (1991–1993) and long-term outcomes at the end of follow-up (1999–2001). Data from the ECRHS study.

Long-term outcomes (measure of association)	Estimate* [95%CI]	p-value
Hospitalization** (RR)^†^	1.31 [1.24, 1.39]	<0.001
N. of asthma attacks^‡^ (RE)^§^	1.46 [1.30, 1.63]	<0.001
Use of controller drugs^‡^ (RE)^§^	1.25 [1.21, 1.29]	<0.001
At least one working day lost^‡^ (RE)^§^	1.25 [1.16, 1.35]	<0.001

RR, adjusted rate ratio; RE, adjusted ratio of expected values.

* for one-unit increase in STS.

** at least one emergency department visit and/or hospital admission for breathing problems during the 9-yr follow-up (between the ECRHS I and II).

^†^ adjusted for gender, age, BMI and smoking habits at ECRHS I.

^‡^ in the past 12 months at ECRHS II.

^§^ adjusted for gender, age, BMI and smoking habits at baseline, and length of the follow-up.

### Replicability of STS

**Fig 3:**
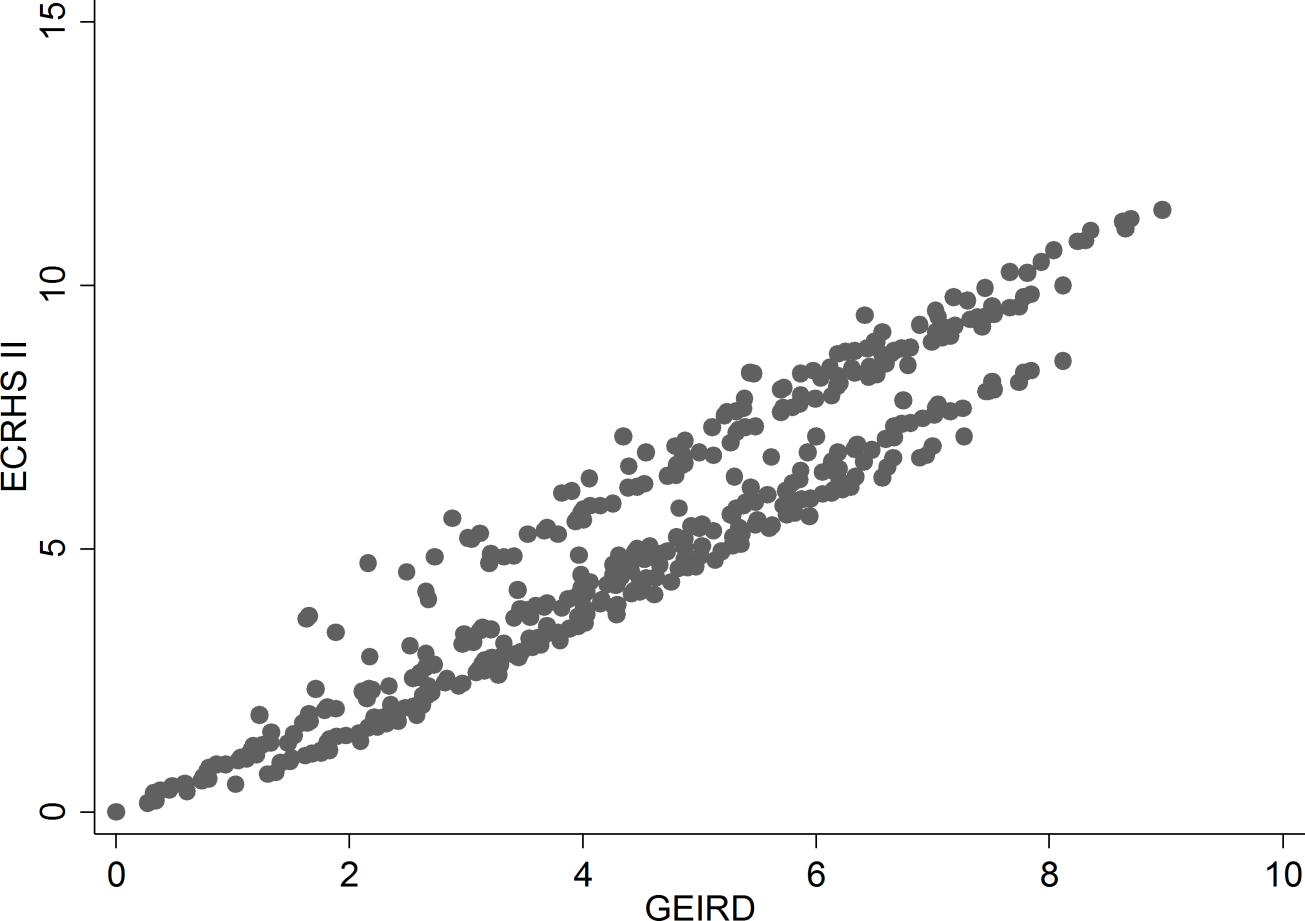
Relationship between the Symptom frequency and anti-asthmatic Treatment intensity Score (STS) computed by using the weights from the GEIRD data (horizontal axis) and STS computed by using the weights from the ECRHS II data (vertical axis).

In the ECRHS cohort, 1,327 subjects with ever asthma had provided complete information on STS at the ECRHS II (Table D in S1 File). The STS scores computed from the GEIRD and ECRHS II data were comparable (Fig 3), the Lin’s coefficient being equal to 0.95 (p-value<0.0001), which indicates an excellent replicability.

## Discussion

The main results of the present study are the following:

1. Lung function variables were weakly correlated with respiratory symptoms and anti-asthmatic treatment in adults. Therefore, lung function, symptom and treatment variables should not be summarized in one dimensional score of asthma severity.
2. A continuous measure of asthma severity has been devised, which summarizes the frequency of respiratory symptoms and the intensity of anti-asthmatic treatment. This score has been proved to be a valid measure of asthma severity in adults and it seems to be replicable in adult populations from different European countries. Moreover, a parametric modelling approach can be adopted in analyzing this score.

### Different dimensions of asthma severity

We investigated which scores could summarize the different dimensions of asthma severity in adult patients, i.e. the frequency of respiratory symptoms, the intensity of anti-asthmatic treatment and lung function. Since the list of symptoms described in the GINA guidelines is claimed to be incomplete [29], a larger set of respiratory symptoms was considered in the present analyses.

In order to create summary measures of asthma severity, the redundancy of information in the considered variables is essential. A correlation between respiratory symptoms and lung function is expected because wheezing, tightness in the chest and cough are most likely to be obstructive symptoms. However, other respiratory symptoms, such as shortness of breath, probably reflect non-obstructive dyspnea [29]. We found a weak correlation between our set of symptom and anti-asthmatic treatment variables, and the two lung function measures. This fact is in agreement with the results from other studies, which have shown a modest association between lung function decline and increased respiratory symptoms [30–31]. In addition, only 5% and 8% of the variance of FEV_1_% predicted and FEV_1_/FVC, respectively, is explained by a common, unobserved factor found in the EFA analysis. When EFA was repeated without the lung function measures, the unobserved factor explains more than 84% of the common variance of the symptom and treatment variables. Therefore, combining respiratory symptoms and lung function variables into a single score is not supported by our data, as found in another study in which lung function had only a small impact on the categorization of asthma severity [32].

MFA helps to remove the redundant information in symptom and treatment variables. We identified two components that explain a high proportion of the total variance (Table C and Figure A in S1 File). The first component can be interpreted as a measure of asthma severity in adult subjects because all weights connected to the frequency of respiratory symptoms and the intensity of anti-asthmatic treatment have a positive sign. The second component separates the patients with the maximum intensity of treatment but no symptoms, from the individuals with no treatment but the maximum frequency of symptoms, because the weights of the treatment and symptom variables have an opposite sign. Therefore, the second component should be a measure of asthma control. In fact, recent guidelines report that asthma control refers to the extent to which the disease manifestations have been reduced or removed by treatment [33]. Despite being different clinical constructs, asthma severity and asthma control are related. In fact, we found a weak negative correlation between STS and the 5-item ACT score (Pearson’s coefficient = -0.47, p-value<0.0001).

### STS and asthma severity

In several epidemiological studies, measures of asthma severity are computed as the sum of symptoms (i.e. equal weights are assigned to each component variable) [34–35]. STS makes it possible to weight the contribution to asthma severity of each symptom and anti-asthmatic treatment in respect to these simple scores. The self-reported intensity of anti-asthmatic treatment has the highest weight in STS. Asthma medications reflect the physician assessment of a patient's underlying severity. In fact, the utilization of a categorical classification of treatment intensity as an approximate index of asthma severity is suggested in population studies where clinical data on disease severity are lacking [36]. Among respiratory symptoms, wheezing has the highest weight in the STS equation, followed by asthma attacks. In fact, different studies have shown that wheezing (alone or in combination with other symptoms) is the most common physical finding in adult asthma [37–39].

The use of continuous scores is recommended in epidemiological studies [18]. In fact, asthma symptoms exist as a continuum in a population [19], and data supporting the existence of one (or more) cut-off points that discriminate subjects into severe (or different levels of severity) and not-severe asthma, are somewhat arbitrary. Furthermore, continuous measures make it possible to increase the power in association analyses, and this fact is particularly important when the sample size is small [19]. Moreover, the power of an association analysis further increases by using a parametric modelling approach for STS, since it is possible to assume a known theoretical distribution of the score [40].

STS is a useful measure of asthma severity in population studies because its validity and replicability, which are two fundamental characteristics of any measurement procedure [41], were demonstrated. Concurrent validity was proved by evaluating the relation between STS and an alternative classification of asthma severity, based on the GINA guidelines. However, the predictive validity is a stronger criterion for validating a measuring instrument [42], as compared to the concurrent validity, because a gold standard for asthma severity does not currently exist. Accordingly, strong positive relationships were observed between an increasing level of STS and the worsening of different long-term outcomes. Moreover, we found a very small variation in the STS weights, when they were computed by using the data from geographically and culturally different European populations (ECRHS). This variation in STS weights (Fig 3) can be attributed to a combination of differences between the clinical questionnaires used in the GEIRD and ECRHS II studies (see “Strengths and weakness of the study” paragraph) and between the characteristics of the GEIRD and ECRHS populations (Table D in S1 File).

### Lung function and asthma severity

Pre-bronchodilator FEV_1_% predicted and FEV_1_/FVC are used as objective measures of respiratory health. The use of FEV_1_ is recommended to determine the severity of airflow obstruction, and FEV_1_/FVC to confirm an obstructive defect [43]. Lung function measurements are important because patients, especially those who have frequent exacerbations, could have a poor perception of the severity of their symptoms [44]. In addition, people who have a sedentary lifestyle might not experience bothersome symptoms even if they have low lung function [33]. However, lung function tests alone are not sufficient, and the understanding of patient’s symptom severity is equally important.

### Strengths and weakness of the study

The main strength of the present analysis is that our cases of asthma underwent an accurate phenotyping by means of an extended clinical interview and lung function tests [20]. Moreover, the data were collected in patients who had been identified from the general population, rather than from clinically selected groups, which should guarantee that our sample encompasses a wide spectrum of disease severity.

A few caveats should be taken into account when interpreting our results. Although our patients were identified from large samples of subjects from the general population, the number of asthma cases was relatively small. However, our estimates do not change when computed from a larger number of patients selected from the European population. There are some differences between the clinical questionnaires used in the GEIRD and ECRHS II studies. In particular, in the ECRHS II questionnaire, “wheezing” and “worsening of respiratory symptoms” variables refer to the “presence of wheezing or whistling in the past 12 months” and “having at least one emergency department visit or hospital admission for breathing problems in the past 12 months”, respectively. Finally, the age range of the GEIRD (20–64 yrs) and ECRHS (28–57 yrs) cases of asthma are not perfectly overlayable.

## Conclusions

Lung function, symptom and treatment variables seem to represent different dimensions of asthma severity in adults with ever asthma. Therefore, we propose STS as a continuous measure of the frequency of respiratory symptoms and the intensity of anti-asthmatic treatment, to be used in epidemiological studies. This score has been proved to be a valid and replicable measure of asthma severity and its distribution can be approximated to some known parametric distribution, such as a Zero-inflated Gamma.

## Supporting information

S1 File **Table A—**Mixed correlation matrix of the candidate variables. **Table B—**Factor weights, uniqueness and the Kaiser-Meyer-Olkin (KMO) measure for the first factor at EFA, with and without the lung function variables. **Table C—**Mean, 95% confidence interval and coefficient of variation (CV) ** of the maximum eigenvalue, proportion of variance accounted for by each component and weights. **Table D—**Main characteristics of the cases of asthma from the ECRHS study. **Figure A—**Scree plot of eigenvalues from (a) Exploratory Factor Analysis (EFA) and (b) Multiple Factor Analysis (MFA).
(DOCX)

S1 Data Minimal data set to replicate the reported study findings. (XLSX)

## Abbreviations

95%CI: 95% confidence interval
ACQ: Asthma control questionnaire
AHR: Airflow hyperresponsiveness
BMI: Body mass index
COPD: Chronic obstructive pulmonary disease
ECRHS: European Community Respiratory Health Survey
EFA: Exploratory factor analysis
GEIRD: Gene Environment Interactions in Respiratory Diseases
GINA: Global INitiative for Asthma
IQR: Interquartile range
MFA: Multiple factor analysis
RES: Ratio of expected scores
SD: Standard deviation
SoB: Shortness of breath
STS: Symptom frequency and anti-asthmatic Treatment intensity Score
RR: Adjusted rate ratio
RE: Adjusted ratio of expected values
