# Supplementary material for: Assessment of asthma severity in adults with ever asthma: A continuous score
Source: PLoS One. 2017 May 18;12(5):e0177538. doi: 10.1371/journal.pone.0177538 (PMC5436664; doi:10.1371/journal.pone.0177538)
Supplement: S1 File — Table A—Mixed correlation matrix of the candidate variables. Table B—Factor weights, uniqueness and the Kaiser-Meyer-Olkin (KMO) measure for the first factor at EFA, with and without the lung function variables. Table C—Mean, 95% confidence interval and coefficient of variation (CV) ** of the maximum eigenvalue, proportion of variance accounted for by each component and weights. Table D—Main characteristics of the cases of asthma from the ECRHS study. Figure A—Scree plot of eigenvalues from (a) Exploratory Factor Analysis (EFA) and (b) Multiple Factor Analysis (MFA). (DOCX) [file pone.0177538.s001.docx]

**Supporting information file**

**Assessment of asthma severity in adults with ever asthma: a continuous score.**

Lucia Calciano^1*^, Angelo Guido Corsico^2^, Pietro Pirina^3^, Giulia Trucco^4^, Deborah Jarvis^5^, Christer Janson^6^, Simone Accordini^1^

^1^ Unit of Epidemiology and Medical Statistics, Department of Diagnostics and Public Health, University of Verona, Verona, Italy

^2^ Division of Respiratory Diseases, IRCCS “San Matteo” Hospital Foundation, University of Pavia, Pavia, Italy

^3^ Institute of Respiratory Diseases, University of Sassari, Sassari, Italy

^4^ Department of Public Health and Pediatrics, University of Turin, Turin, Italy

^5^ National Heart & Lung Institute, Imperial College, London, United Kingdom

^6^ Department of Medical Sciences, Uppsala University, Uppsala, Sweden

* Corresponding author

E-mail: [lucia.calciano@univr.it](mailto:lucia.calciano@univr.it) (LC)

**Methods**

**Individual scores of asthma severity**

In MFA, the individual score for a given component $(S_{i}: i=1,\ldots,n)$ was calculated as a linear combination of the $m (\leq9)$ retained variables ($X_{j}: j=1,\ldots,m$) by the following equation:

$$S_{i}=a_{1}{(X}_{1i}-\mu_{1})+a_{2}{(X}_{2i}-\mu_{2})+\ldots+a_{\mathrm{mi}}{(X}_{\mathrm{mi}}-\mu_{m})$$

where $a_{j}$ and $\mu_{j}$ are the weights (component loadings) and the mean of X_j_, respectively.

The score was reported on a 0-10 continuous scale by the following transformation:

$$\tilde{S}_{i}=10\times\frac{S_{i}- min}{max-min}$$

where$min=-\sum_{j=1}^{m} a_{j}\mu_{j}$ and $max=\sum_{j=1}^{m} a_{j}{max(x}_{\mathrm{ij}})-\sum_{j=1}^{m} a_{j}\mu_{j}$ are the theoretical minimum and maximum, respectively.

The final equation is the following:

$$\tilde{S}_{i}=10\times\frac{\sum_{j=1}^{m} a_{j}x_{\mathrm{ij}}}{\sum_{j=1}^{m} a_{j}{max(x}_{\mathrm{ij}})}$$

Therefore, the score is not connected to the mean of the single variables.

**Results**

**Dimensionality reduction procedure**

Only one factor with eigenvalue >1 (mean of the eigenvalues) was extracted at EFA **(Fig A in S1 File)**, which accounted for 84% of the total variance, and for 5% and 8% of the variance in pre-bronchodilator FEV_1_% predicted (uniqueness = 0.95) and FEV_1_/FVC (uniqueness = 0.92), respectively. The adequacy of the fitted model was good (overall KMO=0.82), even if the KMO of pre-bronchodilator FEV_1_% predicted and FEV_1_/FVC was equal to 0.63 and 0.70, respectively. In the model without lung function variables, the overall KMO was equal to 0.84 and there was more than a 7% increase in the total variance accounted for by the first factor. **Table B in S1 File** reports the factorial solutions (i.e. factor weights, uniqueness and KMO) with and without the lung function variables.

Therefore, only the 9 variables regarding symptom frequency and anti-asthmatic treatment intensity represent the same dimension of asthma severity and they were considered in the dimensionality reduction procedure.

Two components with eigenvalues >0.37 (mean of the eigenvalues), which account for 41% and 19% of the total variance in the 9 observed variables, respectively, were extracted at MFA even if the scree plot did not suggest a natural break between high and low eigenvalues (**Fig A in S1 File**). Only the first MFA component was considered as a score of asthma severity because all the weights had the positive sign, as expected for a measure of disease severity*.* The MFA solution for the first two components (i.e. maximum eigenvalue, proportion of the variance accounted for by each principal component and component loadings), which was obtained by using a bootstrap algorithm with 50,000 replications, is reported in **Table C in S1 File.** The MFA weights were equal to those of both canonical correlation analysis^[[1]](#footnote-1)^ and nonmetric canonical correlation analysis^[[2]](#footnote-2)^ (results not shown). This second analysis was also used to check the equidistance across categories of the selected variables. Therefore, the use of variables on a quantitative scale seems to be an acceptable choice.

**Table A in S1 File.** Mixed correlation matrix of the candidate variables^†^.

|  | Wheezing**^*^** | Asthma attacks**^*^** | Tightness in chest**^*^** | SOB at rest**^*^** | SOB after strenuous activity**^*^** | SOB at night time**^*^** | Chronic bronchitis | Worsening of respiratory symptoms **^*^** | Treatment**^*^** | FEV_1_% predicted | | FEV_1_/FVC% |  |
| --- | --- | --- | --- | --- | --- | --- | --- | --- | --- | --- | --- | --- | --- |
| Wheezing**^*^** | | 1 |  |  |  |  |  |  |  |  |  |  | |
| Asthma attacks**^*^** | | 0.631 | 1 |  |  |  |  |  |  |  |  |  | |
| Tightness in chest**^*^** | | 0.555 | 0.538 | 1 |  |  | Polychoric/ tetrachoric | |  |  | Polyserial | | |
| SOB at rest**^*^** | | 0.477 | 0.534 | 0.638 | 1 |  |  |  |  |  |  |  | |
| SOB after strenuous activity**^*^** | | 0.499 | 0.417 | 0.512 | 0.407 | 1 |  |  |  |  |  |  | |
| SOB at night time**^*^** | | 0.434 | 0.514 | 0.756 | 0.652 | 0.436 | 1 |  |  |  |  |  | |
| Chronic bronchitis | | 0.368 | 0.212 | 0.450 | 0.388 | 0.394 | 0.455 | 1 |  |  |  |  | |
| Worsening of respiratory symptoms**^*^** | | 0.496 | 0.299 | 0.566 | 0.463 | 0.365 | 0.404 | 0.376 | 1 |  |  |  | |
| Treatment**^*^** | | 0.414 | 0.574 | 0.302 | 0.312 | 0.341 | 0.331 | 0.181 | 0.329 | 1 |  |  | |
| FEV_1_% predicted | | 0.231 | 0.150 | 0.095 | 0.126 | 0.150 | 0.143 | 0.018 | 0.121 | 0.138 | 1 | Pearson | |
| FEV_1_/FVC% | | 0.269 | 0.184 | 0.168 | 0.170 | 0.221 | 0.123 | 0.051 | 0.155 | 0.236 | 0.440 | 1 | |

^†^ polychoric correlation between two ordinal variables, tetrachoric correlation between two dichotomous variables, Pearson moment correlation between two continuous variables, and polyserial correlation if one variable is categorical and the other continuous.

**^*^** in the past 12 months.

**Table B in S1 File.** Factor weights, uniqueness and the Kaiser-Meyer-Olkin (KMO) measure for the first factor at EFA, with and without the lung function variables.

|  | **Model with lung function variables** | | | **Model without lung function variables** | | |
| --- | --- | --- | --- | --- | --- | --- |
|  | **Factor**  **weights** | **Uniqueness** | **KMO** | **Factor**  **weights** | **Uniqueness** | **KMO** |
| Wheezing**^*^** | 0.736 | 0.458 | 0.856 | 0.724 | 0.476 | 0.857 |
| Asthma attacks**^*^** | 0.717 | 0.486 | 0.777 | 0.717 | 0.487 | 0.776 |
| Tightness in chest**^*^** | 0.827 | 0.316 | 0.829 | 0.836 | 0.301 | 0.836 |
| SOB at rest**^*^** | 0.729 | 0.469 | 0.914 | 0.733 | 0.463 | 0.914 |
| SOB after strenuous activity**^*^** | 0.618 | 0.618 | 0.934 | 0.613 | 0.624 | 0.930 |
| SOB at night time**^*^** | 0.765 | 0.414 | 0.821 | 0.773 | 0.402 | 0.831 |
| Chronic bronchitis | 0.508 | 0.742 | 0.878 | 0.519 | 0.731 | 0.893 |
| Worsening of  respiratory symptoms**^*^** | 0.613 | 0.624 | 0.811 | 0.615 | 0.622 | 0.809 |
| Treatment**^*^** | 0.528 | 0.722 | 0.773 | 0.517 | 0.733 | 0.776 |
| FEV_1_% predicted | 0.230 | 0.947 | **0.632** | - | - | - |
| FEV_1_/FVC% | 0.290 | 0.916 | **0.696** | - | - | - |

**^*^** in the past 12 months.

**Table C in S1 File.** Mean, 95% confidence interval and coefficient of variation (CV) ^†^ of the maximum eigenvalue, proportion of variance accounted for by each component and weights

|  | **Component 1** |  | **Component 2** |  |
| --- | --- | --- | --- | --- |
|  | Mean [95%CI] | CV | Mean [95%CI] | CV |
| Maximum eigenvalue (λ_1_) | 1.39 [1.30, 1.48] | 0.000 | 0.63 [0.55, 0.72] | 0.000 |
| Proportion of variance (%) | 41.46 [37.62, 45.32] | 0.000 | 18.81 [16.31, 21.45] | 0.000 |
| Weights |  |  |  |  |
| Wheezing**^*^** | 0.554 [0.479, 0.636] | 0.000 | 0.671 [0.546, 0.808] | 0.000 |
| Asthma attacks**^*^** | 0.452 [0.386, 0.521] | 0.000 | 0.190 [0.044, 0.330] | 0.002 |
| Tightness in chest**^*^** | 0.255 [0.212, 0.300] | 0.000 | 0.345 [0.255, 0.438] | 0.001 |
| SOB at rest**^*^** | 0.171 [0.126, 0.214] | 0.001 | 0.180 [0.099, 0.260] | 0.001 |
| SOB after strenuous activity**^*^** | 0.244 [0.193, 0.296] | 0.000 | 0.239 [0.132, 0.344] | 0.001 |
| SOB at night time**^*^** | 0.201 [0.152, 0.249] | 0.001 | 0.231 [0.138, 0.323] | 0.001 |
| Chronic bronchitis | 0.144 [0.090, 0.201] | 0.001 | 0.199 [0.093, 0.301] | 0.001 |
| Worsening of respiratory symptoms**^*^** | 0.182 [0.131, 0.233] | 0.001 | 0.172 [0.078, 0.266] | 0.001 |
| Treatment**^*^** | 0.709 [0.668, 0.757] | 0.000 | -0.674 [-0.725, -0.627] | 0.000 |

^†^ obtained by a bootstrap estimating procedure.

**^*^** in the past 12 months.

**Table D in S1 File.** Main characteristics of the cases of asthma from the ECRHS study included in the predictive validity and replicability analyses of STS.

| **Main characteristic** | **Sub-characteristic** | **Predictive validity analysis**  **(N =1,097)** | **Replicability analysis**  **(N = 1,327)** |
| --- | --- | --- | --- |
| Females, % |  | 56.8 | 57.3 |
| Age (years), mean ± sd |  | 33.8 ± 7.2 | 42.6 ± 7.2 |
| Smoking habits, % | Never smoker | 48.9 | 44.9 |
|  | Past smoker | 21.0 | 26.4 |
|  | Current smoker | 30.2 | 28.7 |
| BMI, median (interquartile range) |  | 23.3 (21.2-25.8) | 24.9 (22.4-28.1) |
| Wheezing^*^, % |  | 71.5 | 63.1 |
| Asthma attacks^*^, % | None | 54.9 | 70.5 |
|  | 1-11 attacks | 35.3 | 24.0 |
|  | ≥ 12 attacks | 9.8 | 5.6 |
| Tightness in chest^*^, % |  | 45.3 | 37.4 |
| SOB at rest^†^, % |  | 27.8 | 21.3 |
| SOB after strenuous activity^*^, % |  | 50.3 | 42.4 |
| SOB at night time^*^, % |  | 31.4 | 22.2 |
| Chronic bronchitis, % |  | 26.5 | 21.3 |
| Emergency department visits or hospital admissions for breathing problems^*^, % |  | 31.2 | 15.4 |
| Treatment^*^, % | None | 38.6 | 48.6 |
|  | GINA step 1 - only relievers | 28.1 | 17.0 |
|  | GINA step 1 - controllers | 15.6 | 13.7 |
|  | GINA steps ≥ 2 | 17.7 | 20.7 |
| Pre-bronchodilator FEV_1_ % predicted, mean ± sd |  | 99.0 ± 15.7 | 97.7 ± 17.0 |
| Pre-bronchodilator FEV_1_/FVC, mean ± sd |  | 78.8 ± 9.0 | 76.2 ± 8.6 |

SOB: shortness of breath.

**^*^** in the past 12 months.

**Fig A in S1 File.** Scree plot of eigenvalues from (a) Exploratory Factor Analysis (EFA) and (b) Multiple Factor Analysis (MFA). The dashed lines indicate the average of the eigenvalues.

|  |  |
| --- | --- |

1. Hotelling, H., 1936*:* Relations between two sets of variants. Biometrika, 28, 321-377*.* [↑](#footnote-ref-1)
2. *A.* Gifi*.* Nonlinear Multivariate Analysis. Department of Data Theory FSW/RUL, Leiden, The Netherlands*,* 1981*.* [↑](#footnote-ref-2)
